# Homozygous splice-site variant in *ENPP1* underlies generalized arterial calcification of infancy

**DOI:** 10.1186/s12887-024-05123-0

**Published:** 2024-11-13

**Authors:** Hafiza Noor Ul Ayan, Yvonne Nitschke, Abdul Razzaq Mughal, Holger Thiele, Naveed Altaf Malik, Ijaz Hussain, Syed Muhammad Ijlal Haider, Frank Rutsch, Jeanette Erdmann, Muhammad Tariq, Zouhair Aherrahrou, Ilyas Ahmad

**Affiliations:** 1 Institute for Cardiogenetics, University of Lübeck, Lübeck, 23562 Germany; 2 National Institute for Biotechnology and Genetic Engineering College, Pakistan Institute of Engineering and Applied Sciences (NIBGE-C, PIEAS), Faisalabad, 38000 Pakistan; 3 DZHK (German Research Center for Cardiovascular Research), Partner Site Hamburg/Lübeck/Kiel, Lübeck, 23562 Germany; 4 Department of General Pediatrics, Muenster University Children’s Hospital, Muenster, 48149 Germany; 5 Faisalabad Institute of Cardiology, Faisalabad, 38000 Pakistan; 6 Cologne Center for Genomics (CCG), University of Cologne, Faculty of Medicine and University Hospital Cologne, Cologne, 50931 Germany; 7 Peshawar Institute of Cardiology, Peshawar, 25000 Pakistan

**Keywords:** GACI, *ENPP1*, Splice site variant

## Abstract

ENPP1 (ectonucleotide pyrophosphatase/phosphodiesterase 1) plays a critical role by converting extracellular ATP to AMP, generating extracellular PPi, a potential inhibitor of calcification. Pathogenic variants in the *ENPP1* cause generalized arterial calcification of infancy (GACI [OMIM 208000]). GACI, is an ultra-rare disease characterized by early-onset calcification of large and medium-sized arteries, leading to severe cardiovascular complications such as heart failure, pulmonary stenosis (PS), hypertension, and more. In this study, we report a novel homozygous splice-site pathogenic variant in *ENPP1* (NM_006208, c.2230 + 5G > A; p.Asp701Asnfs*2) residing in C-terminal nuclease-like domain (NLD) of ENPP1 protein in a Pakistani family diagnosed with severe valvular PS and mild right ventricular hypertrophy (RVH). cDNA assays confirmed the skipping of exon 21, and the splice product underwent nonsense-mediated decay. Functional studies on fibroblasts from the patient demonstrated increased calcification and decreased enzymatic activity of ENPP1, recapitulating the hallmarks of GACI. By combining genetic analysis with the in vitro study, we substantiate that *ENPP1*:c.2230 + 5G > A variant is pathogenic, underscoring its role in the development of GACI.

**Supplementary Information:**

The online version contains supplementary material available at 10.1186/s12887-024-05123-0.

## Introduction

ENPP1, the founding member of ectonucleotide pyrophosphatase phosphodiesterase (ENPP) family of proteins, is a type II transmembrane glycoprotein encoded by the *ENPP1* gene (MIM 173335). Orginally described as the plasma cell-differentiation antigen PC-1, ENPP1’s importance extends to purinergic signaling system and, essential for a wide array of physiological functions in mammals including regulation of cardiovascular, neurological, immunological, musculoskeletal, and hormonal functions [1, 2]. ENPP1 is expressed on the cell surface in mineralizing cells, such as osteoblasts and chondrocytes, lymphoid organs and catalyzes the hydrolysis of extracellular adenosine triphosphate (ATP) into adenosine monophosphate (AMP) and inorganic pyrophosphate (PPi) [3, 4]. PPi, serves as a potent antimineralization agent that binds to nascent hydroxyapatite crystals and inhibits their growth to prevent pathological calcification [5]. In addition to its role in preventing vascular calcification, PPi plays a critical role in inhibiting ectopic calcification through its regulation of chondrogenesis, collagen I expression, and synthesis [4–6]. Thus, the generation of PPi makes ENPP1 an important modulator of vascular calcification and bone development in mammals [7].

*ENPP1* gene maps to chromosome 6q23.2, spans 25 exons and encodes ecto-nucleotide pyrophosphatase/phosphodiesterase 1 (ENPP1), a protein of 925 amino acids [8, 9]. Structurally complex, ENPP1 comprises functional domains including N-terminal cytoplasmic domain (CD), transmembrane domain (TM), tandem somatomedin-B-like domains (SMB1-2), a phosphodiesterase catalytic domain (PCD), L1 and L2 linkers, and a C-terminal nuclease-like domain (NLD) [9]. These domains collectively contribute to the diverse functions of ENPP1 in various physiological processes. Notably, the CD and TM domains are involved in the protein’s localization within the plasma membrane and facilitating dimerization through disulfide bond formation [10–12]. The SMB domains were previously thought to be involved in homodimerization of ENPP1 protein [13], however, a study by Jansen et al. shows that it mediate trafficking to the plasma membrane [10]. PC domain, as the name implies, is the primary functional domain responsible for hosting the common pyrophosphatase/phosphodiesterase activity and is homologous to the alkaline phosphatases family [14]. The L1 and L2 linkers are involved in maintaining tight interdomain interactions [15]. The NL domain has no catalytic activity itself but is required for the translocation of ENPP1 from the endoplasmic reticulum to the Golgi-apparatus and is critical for the proper folding of protein [16].

Pathogenic variants in *ENPP1* are associated with several genetic disorders, including three autosomal recessive conditions: generalized arterial calcification of infancy (GACI [MIM 208000]), autosomal recessive hypophosphatemic rickets (ARHR2 [MIM 613312]), and pseudoxanthoma elasticum (PXE [MIM 264800]), along with autosomal dominant Cole disease [MIM 615522] and insulin resistance and type 2 diabetes. Biallelic loss-of-function variants in *ENPP1* lead to NPP1 (nucleotide pyrophosphatase/phosphodiesterase 1) deficiency, a rare disorder characterized by low PPi levels, resulting in excessive soft tissue calcification, arterial stenoses, and bone hypomineralization [17, 18].

GACI is a rare and severe autosomal-recessive disorder marked by calcification of the internal elastic lamina, fibrotic myointimal proliferation of muscular arteries, and subsequent arterial stenosis. Biallelic inactivating mutations in the *ENPP1* gene lead to the loss of function of the ENPP1 protein, resulting in low PPi levels dysregulation and subsequent ectopic calcification of arterial walls. It is estimated to occur at one in 200,000 pregnancies, with a carrier frequency of one in 223 individuals [17]. Individuals with GACI may experience breathing difficulty, edema, cyanosis, hypertension, cardiomegaly and ultimately heart failure [19]. In addition to cardiovascular phenotype, GACI can also lead to the deposition of calcium and other minerals in elastic fibers of connective tissues. This can result in a phenotype similar to pseudoxanthoma elasticum. Some individuals also manifest periarticular calcifications, ARHR2, cervical spine fusion, and hearing loss [19]. Unfortunately, the disease prognosis is usually very poor, with approximately 50% of affected individuals not surviving beyond the first six months of life due to severe cardiovascular complications [20–22]. However, survival into third and fourth decade of life has also been reported. GACI can be classified into type 1 and type 2 based on the underlying genetic cause. GACI type 1 (MIM 208000) is primarily caused by pathogenic variants in the *ENPP1* gene, accounting for 67–75% of all cases [19, 23]. GACI type 2 (MIM 614473), on the other hand, is caused by pathogenic variants in the *ABCC6* gene, responsible for 9–10% of all GACI cases [19, 22, 24, 25]. The underlying cause for the rest of cases remains unknown.

In the present study, we report a novel homozygous splice-site variant in *ENPP1* (NG_008206.1(NM_006208.3):c.2230 + 5G > A), in a Pakistani patient diagnosed with severe valvular pulmonary stenosis (PS) and mild right ventricular hypertrophy (RVH). The variant was investigated for its functional impact on the ENPP1 protein, enzymatic activity and regulation of calcification, providing evidence that the biallelic loss of its protein function leads to GACI in humans.

## Materials and methods

### Human subjects

**Fig. 1:**
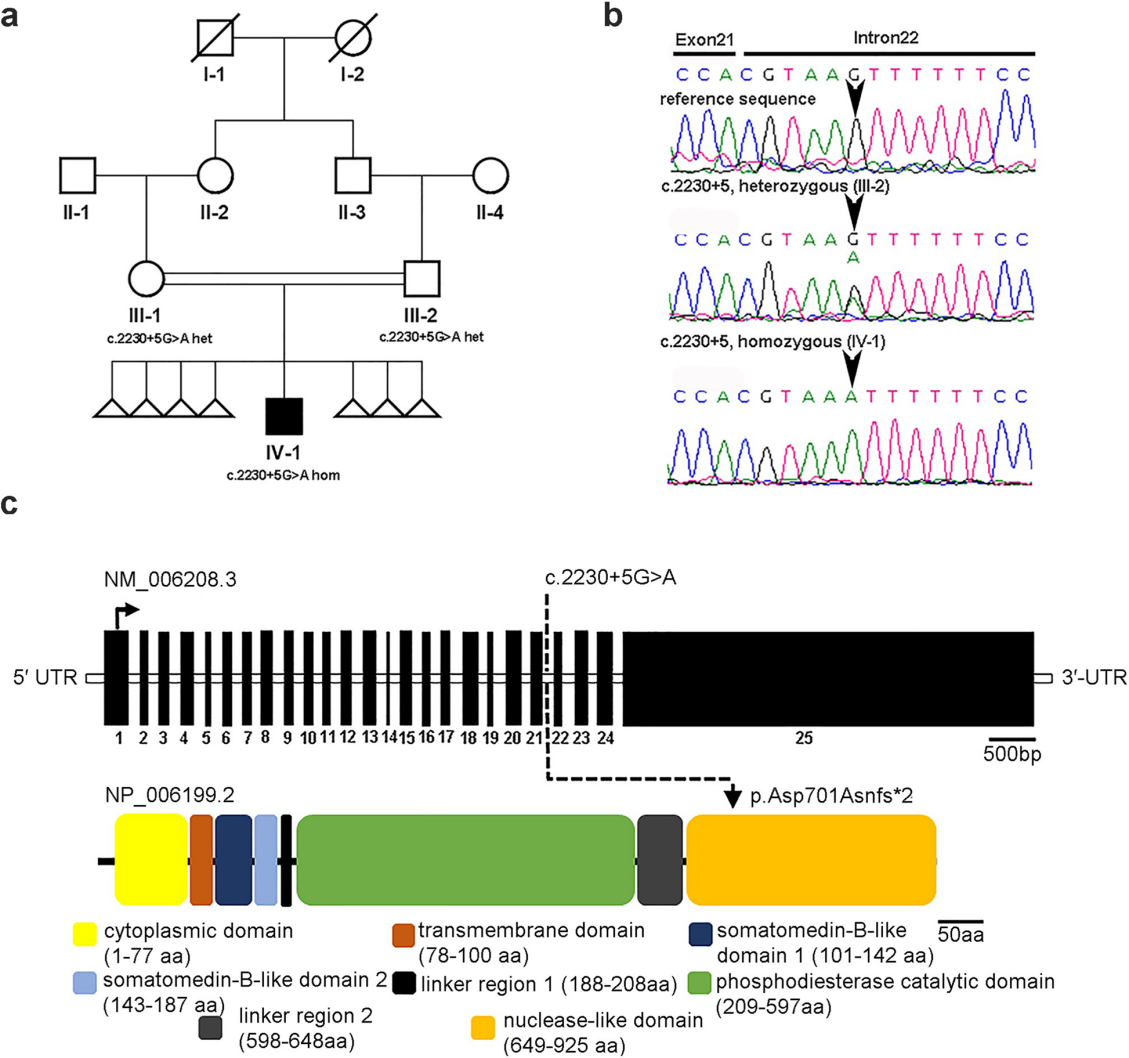
A homozygous splice site (c.2230 + 5G > A; p.Asp701Asnfs*2) in a family. **a** The family pedigree illustrates a patient carrying a pathogenic variant in *ENPP1* (c.2230 + 5G > A). Open symbols represent unaffected individuals, filled symbols represent affected individual, triangles represent spontaneous abortions, and symbols with a diagonal line indicate deceased individuals. The genotypes of the *ENPP1* variant are shown below each examined member. **b** Representative sequence electropherograms show the wild-type sequence and the (c.2230 + 5G > A) heterozygous (III-2) and homozygous (IV-1) sequences. **c** Schematic of the human *ENPP1* gene and protein domain structure. The ENPP1 protein consists of the N-terminal cytoplasmic domain (CD), transmembrane domain (TM), tandem somatomedin-B-like domains (SMB1-2), a phosphodiesterase catalytic domain (PCD), L1 and L2 linkers, and a C-terminal nuclease-like domain (NLD). Position of (c.2230 + 5G > A; p.Asp701Asnfs*2) is indicated

The proband, a 5 years old male, was a pediatric patient at the Faisalabad Institute of Cardiology (FIC), Faisalabad, Pakistan, diagnosed with PS and RVH. The proband’s family members were invited from the Sargodha district of Punjab province, Pakistan, and a detailed family history was obtained (Fig. 1a). Blood samples from the patient and his unaffected parents (III-1 and III-2) were collected in BD vacutainers® (BD-Plymouth, Plymouth, UK) containing ethylenediaminetetraacetic acid (EDTA).

### Genetic work up

Genomic DNA (gDNA) was extracted from the whole blood using standard protocols. The details of the whole exome sequencing procedure have been previously described [26, 27]. In brief, exonic and adjacent intronic sequences were enriched from genomic DNA using the Agilent SureSelect Human All Exon V6 r2 kit (60 Mb; Agilent Technologies, Santa Clara, USA) and were run on an Illumina HiSeq 4000 system (Illumina, San Diego, USA) with a paired end 2 × 75 bp protocol. Sequencing data were mapped to the human reference genome (hg38/GRCh38 assembly, NCBI) with the Burrows–Wheeler aligner (BWA) algorithm. For downstream processing, different software tools such as Genome Analysis Toolkit (GATK), SAMtools, and Picard were utilized for various steps in the analysis pipeline. The average exome coverage was 77X, with over 96% of exomes having coverage greater than 10X. Data processing, variant calling, annotation, and filtering were conducted using the exome and genome analysis pipeline “VARBANK (v2.25)” (https://varbank.ccg.uni-koeln.de/varbank2/) of the Cologne Center for Genomics (CCG, University of Cologne, Germany). High quality (coverage of more than 6 reads), a minimum quality score of 10, an allele frequency ≥ 75%, rare (minor allele frequency < 0.1%) in the gnomAD database (http://gnomad.broadinstitute.org/), homozygous variants within regions of homozygosity (ROH), and variants that were not annotated in the CCG WES dataset were filtered as previously described [26, 27]. Variants were checked for their presence in two widely used databases, ClinVar and HGMD (Human Gene Mutation Database) and were carefully classified in accordance with the standards set forth by the American College of Medical Genetics and Genomics (ACMG) [28]. Allele frequency was obtained from the Genome Aggregation Database (gnomAD browser: http://gnomad.broadinstitute.org). Probability of exon skipping and activation of cryptic splice sites resulting from the identified splice site variant was estimated using the CRY*P-*SKIP algorithm (http://cryP-skip.img.cas.cz/) [29]. Bi-directional Sanger sequencing was performed for validation and co-segregation of variants.

### Polymerase chain reactions (PCR) and Sanger sequencing

PCR amplification of both genomic DNA (gDNA) and complementary DNA (cDNA) sequences flanking the splice variant site (*ENPP1*; c.2230 + 5G > A) was performed. Specific primer sets were designed using Primer3 software (supplementary Table S1). The PCR and reverse transcription PCR (RT-PCR) products were purified using NucleoSpin™ Gel and PCR Clean-up kit (Macherey–Nagel GmbH & Co. KG, Dueren, Germany). The purified products were subsequently processed for bidirectional Sanger sequencing provided by a commercial service provider (Microsynth AG, Sankt Gallen, Switzerland). All reactions were performed according to the manufacturer’s recommendations. To assess the segregation of the variant, gDNA samples from other family members were utilized. DNASTAR Lasergene v17 (DNASTAR Inc., Madison, USA) software was used to visualize the chromatograms.

### Real-time quantitative PCR (qPCR)

Total RNA was extracted from whole blood samples of the proband and his healthy parents using the PAXgene blood RNA kit (Qiagen, Hilden, Germany) according to the optimized manufacturer’s protocols. RNA concentration and quality was measured using NanoDrop ND-1000 (PEQLAB Biotechnology GmbH, Erlangen, Germany). Complementary DNA (cDNA) was reverse transcribed from total RNA following the previously described method [30]. qPCR was performed using the cDNA derived from the proband, his parents and control (WT-1). For this purpose, a reaction mixture of Luna® Universal qPCR Master Mix (New England Biolabs, UK), 0.25 µM of each primer, and cDNA (transcribed from 15 ng/µL RNA) was prepared. The experiment was performed in three replicates using the 7900HT Fast Real-Time PCR System (Applied Biosystems, Waltham, USA). The cycle threshold (Ct value) was determined by analytical software SDS2.2.2 (Applied Biosystems, Waltham, USA). qPCR quantification was calculated according to the 2^–∆∆Ct^ method [31].

### Cell culture

Dermal fibroblasts from the patient were obtained through a punch biopsy of the skin. The cultured fibroblasts were maintained in DMEM (Dulbecco’s Modified Eagle Medium) from Thermo Fisher Scientific (Waltham, USA), supplemented with 10% Fetal Bovine Serum (FBS), 10 µg/mL Gentamycin, 250 ng/mL Amphotericin B, 100 U/mL Penicillin, and 100 µg/mL Streptomycin. This medium composition was used for passage 0 and passage 1 of the fibroblasts. For further subculturing, the medium was changed, and the cells were cultivated in DMEM, 10% FBS, 250 µM ascorbic acid sodium salt and 2 ng/µL fibroblast growth factor (FGF2). Using the same protocol, a wild type cell line derived from a 28-year-old European healthy female (WT) was cultured as a healthy control. Both the patient-derived fibroblasts and the wild-type control cells were used at the same passage in each experiment, ensuring a consistent comparison between the pathological and control cells.

### Western blotting (WB)

Cultured fibroblasts from the patient and WT were lysed in a lysis buffer containing 1.6 mM MgCl2, 0.2 M Tris (pH 8.1) and 1% Triton X-100. The supernatant of cell lysate was collected, and the protein concentration of each sample was determined using the Lowry method [32] with the DC™ Protein Assay kit (Bio-Rad Laboratories, Hercules, USA). For western blot analysis, triplicates of 30 µg protein samples were resolved on a SDS-PAGE gradient gel (7.5%-12%). The samples were then resolved on SDS-PAGE gradient (7.5%-12%) and proteins were electrophoretically transferred to a polyvinylidene fluoride (PVDF) membrane (Merck KGaA, Darmstadt, Germany). After the transfer, the membrane was blocked in a solution of 5% w/v nonfat dry milk in Tris-buffered saline (TBS) (20 mM Tris, 150 mM NaCl, pH 7.6) for 1 h to prevent nonspecific protein binding. Next, the membrane was probed with primary antibodies, ENPP1 (5342S, Cell Signaling Technology, Danvers, USA) and β-actin (A1978, Sigma-Aldrich Co LLC, Burlington, USA). Afterwards the membrane was incubated with appropriate horseradish peroxidase (HRP)-conjugated secondary antibodies. To visualize the bound antibodies, the membrane was exposed to chemiluminescence (using the Amersham ECL Prime Western Blotting Detection Reagent, Cytiva (Emeryville, USA)). Image processing and capture were performed using software provided by the manufacturer. Images were superposed with GIMP v2.10 software.

### Nucleotide pyrophosphatase/phosphodiesterase (NPP) activity assay

The NPP activity assay was using cell lysates from the patient, WT healthy control cells, and a previously reported GACI patient with a homozygous variant (c.2677G > T, p.E893X, homozygous) [23, 33]. The previously reported GACI patient was included as a positive control in the assay to compare the NPP activity levels with those of our patient and the WT control. NPP activity from the cell lysate was measured by colorimetric assay using a synthetic substrate *P-*nitrophenylthymidine 5’-monophosphate (PNTM) as described previously [33].

### In vitro* calcification assay*

For in vitro calcification assay, fibroblasts from the patient and healthy control (WT) were used. Cells were cultured at a density of 40,000 cells/well in a standard medium of DMEM and 10% FBS. When cells reached 85–90% confluency, half of the wells were left untreated, while in the remaining wells mineralization was induced in vitro by treating the fibroblasts with calcification medium. The calcification medium was prepared using DMEM, 10% FBS, 10 mM ß-glycerophosphate, 50 µg/mL ascorbic acid, and 10 nM dexamethasone, with slight modifications based on a previously published protocol [34]. The medium was replaced twice a week. The assay was repeated four times, and the fibroblasts were regularly observed under an Eclipse TS100 inverted microscope (Nikon Instruments Inc, New York, USA). The experiment was terminated after 23 days.

To detect mineral deposits, half of the wells were fixed in 10% paraformaldehyde (PFA) at 4 °C for 24 h. Cells were then washed twice with 50 mM Tris–HCL (pH 9) and stained with Calcein in the dark for 30 min. After staining, the cells were washed twice with 50 mM Tris–HCL, for 5 min each, and then nuclei of cells were counterstained with a blue-fluorescent DAPI for 15 min in the dark. Following the removal of the stain, cells were covered with 50 mM Tris–HCL wash buffer and observed under compact fluorescence microscope (BZ-X810, Keyence, Neu-Isenburg, Germany). Image processing and capture were performed using software provided by the manufacturer. A Python script was used to estimate Calcein intensity, DAPI intensity, and pixel count. The calcein intensity was normalized to DAPI (Intensity Calcein/DAPI pixel count) as described elsewhere [30].

For calcium quantification, the other half of the wells were treated with 0.6 M HCl at 4 °C. After 24 h, the samples were scraped off, transferred to Eppendorf tubes, and centrifuged (13,000 rpm) for 20 min at 4 °C. A portion of the supernatant was transferred to a fresh tube, and calcium concentration was quantified using a Calcium Assay kit (Randox Laboratories Ltd, Crumlin, UK). The calcium content was then normalized to protein concentration. Microplate data were collected and analysed using BioTek Gen5™ software (Agilent Technologies, Santa Clara, USA).

### Statistical analysis

Data were assessed for normality by Shapiro–wilk test. Values were analyzed by Student’s t-test and two-way ANOVA. Multiple comparisons were selected for two-way ANOVA and Tukey’s test was used to avoid an inflated Type I error rate. Where indicated, error bars represent standard deviation (SD). For all analyses, P values less than 0.05 were considered statistically significant. All statistical tests were performed using Prism v9.3 (GraphPad, San Diego, USA).

## Results

### Clinical history of the proband

The proband was born at full-term after an uncomplicated pregnancy of a consanguineous couple (Fig. 1a). However, at the age of 1 year, he was admitted to the hospital due to symptoms including feeding difficulty, vomiting, irritability and fatigue. Echocardiography revealed anomalies in the valves and chambers of the heart. The patient had severe pulmonary valve stenosis (PS), with a peak pulmonary valve gradient of 76 mmHg and a pulmonary valve annulus diameter of 6.5 mm, which was at least 5.2 mm below the normal range (mean ± SD, 13.3 ± 1.6 mm) [35] based on body surface area (0.45 m^2^). The patient also had a mild pathological increase in right ventricular muscle mass in response to PS, a condition known as RVH. Furthermore, the mother of the proband experienced at least seven miscarriages in the ten years of her married life, with four occurring before the birth of the proband and three occurring after.

### Ex*ome sequencing identifies an intronic variant c.2230* + *5G* > *A in ENPP1*

Exome sequencing was performed on the proband to identify potential pathogenic variant underlying the disease phenotype. Considering the consanguinity of unaffected parents, an autosomal recessive pattern of inheritance was inferred. Variants were filtered based on homozygous runs (ROHs) of ≥ 5 Mb in length. This led to the identification of a novel homozygous splice site variant GRCh38:Chr6: g.131882479G > A [c.2230 + 5G > A (NM_006208)], located in intron 21 of the *ENPP1* (Fig. 1b, c; Fig. S1).The variant was assessed for its impact on *ENPP1* splicing using different computational tools. It was found to have a high median value (0.95) indicating its effect on *ENPP1* splicing, as determined by AdaBoost [36] (ADA, 0.99) and Random Forest (RF, 0.89) [37] algorithms. Given the known association of *ENPP1* with GACI type 1 and cardiovascular phenotypes, this variant is the most likely candidate for the phenotype under investigation. Moreover, this variant was absent from publicly available allele frequency databases, [Genome Aggregation Database (gnomAD), dbSNP (build 155), 1000 Genomes Project], disease specific databases [Clinvar and Human Gene Mutation Database (HGMD)], and in-house datasets of CCG (> 3200 Exome)., In addition, the variant was absent in the 160 exomes of familial cardiac diseases from Pakistan. Based on ACMG guidelines [28] (criteria PVS1, PS3, PM2, PM4, and PP4), the variant was classified as pathogenic. We confirmed the homozygous variant in proband by Sanger sequencing, and co-segregation analysis revealed heterozygous carrier status of both parents (Fig. 1b). The location of variant in the gene and protein structure of ENPP1 is shown in Fig. 1c.

### Variant causes exon skipping and reduction in gene expression

**Fig. 2:**
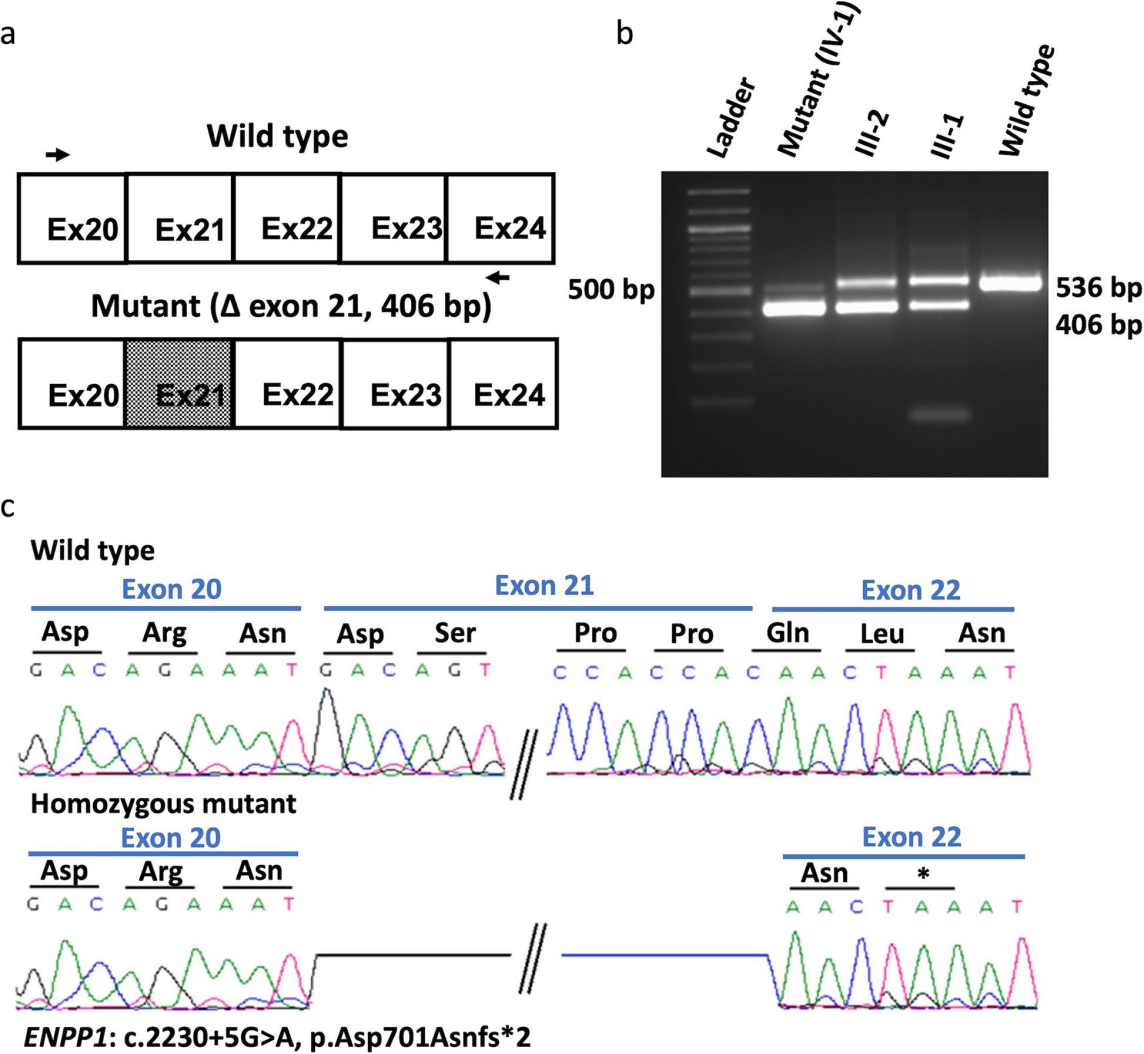
Effect of *ENPP1*: c.2230 + 5G > A variant on the transcipt. **a** PCR products obtained from cDNA of affected individual (IV-1) and heterozygous individuals (III-2, III-1) were separated by electrophoresis on a 1% agarose gel, along with a WT control, using specific primers for the critical ENPP1 region. **b** Schematic of the partial region of ENPP1 wild type transcript (top) with primers locations (black arrows) used for cDNA amplification. The lower panel shows the mis-spliced ENPP1 transcript with exon 21 deletion (shaded region). **c** Sanger traces of the amplicons obtained from wild-type and patient cDNA to verify the presumed skipping of exon 21. The lower panel belongs to smaller, mis-spliced transcript, showing deletion of 130 bp (exon 21 skipping)

**Fig. 3:**
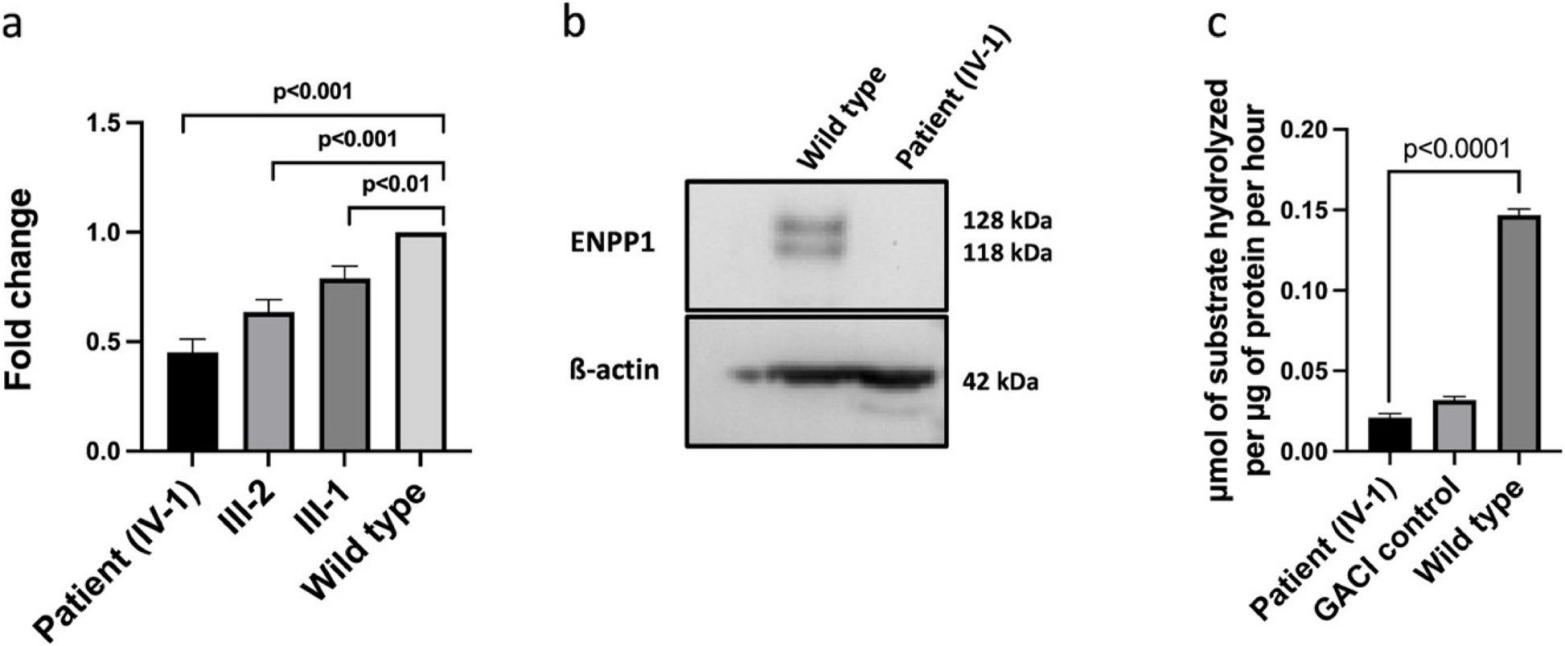
RT-qPCR, Western blot and Nucleotide phosphodiesterase activity assay. **a** Functional ENPP1 levels are 45% lower than normal in the patient (IV-1) with the c.2230 + 5G > A variant. Heterozygous parents have levels at 37% (III-2) and 21% (III-1). Transcript levels were quantified using RT-qPCR on blood-derived RNA (*n* = 3 experiments, triplicate; error bars, SD). Data were first tested for normality using the Shapiro–wilk test. *P-*values were calculated by unpaired, parametric, one-tailed Student’s t-test with Welch correction. **b** Western blot confirms the absence of ENPP1 protein in patient fibroblasts (IV:1) compared to wild type (upper panel). The internal control, ß-actin, is shown in the lower panel. **c** Graph depicts a significant reduction in ENPP1 enzymatic activity in patient fibroblasts compared to wild type. Statistical analysis was performed to determine whether there were significant differences in ENPP1 activity of patient and wild type. Data were assessed for normality by Shapiro–wilk test. *P-*values were calculated by unpaired, parametric, two-tailed Student’s t-test with Welch’s correction

We investigated the effects of nucleotide change (c.2230 + 5G > A) at a conserved 5′ donor splice site of intron 21 on *ENPP1* transcript splicing. The CRY*P-*SKIP analysis revealed a high probability (0.84) of adjacent exon (exon 21) skipping, while the probability of new cryptic splice site activation was 0.16. To validate the splicing alteration, PCR products of patient and parents’ cDNA were amplified using primers upstream (exon 20) and downstream (exon 24) of the variant site (Fig. 2a, supplementary Table 1)). An aberrant product size of 406 bp was observed in the patient’s sample, while the healthy control (WT-1) exhibited the expected product size of 536 bp. Both parents revealed product sizes of 406 bp and 536 bp (Fig. 2b). Sanger sequencing the 406 bp size product confirmed the deletion of exon 21, which spans 130 bp (Fig. 2c). No aberrantly spliced transcripts with cryptic splice site activation were identified in the proband. The skipping of exon 21 leads to a frameshift and thus premature termination of translation (p.Asp701Asnfs*2). This results in the loss of 224 amino acids at the C-terminus of ENPP1 which accounts for approximately one-fourth of the protein. To test whether this activated the nonsense-mediated mRNA decay (NMD) pathway, *ENPP1* transcripts were quantified using qPCR. Compared to the WT, a 55% reduction in *ENPP*1 transcripts was observed in the patient (*p* < 0.001), 37% in the father (*p* < 0.001), and 21% in the mother (*p* < 0.01) (Fig. 3a). This reduction in transcripts suggests that NMD is at work. Additionally, Western blot analysis detected no ENPP1 protein in the fibroblasts of the patient compared to the WT (wrong bracket deleted). ENPP1 monomers are known to migrate as doublets at 118 and 128 kDa on SDS-PAGE [38] (Fig. 3b).

### Variant results in reduced enzymatic activity and increase in calcification of fibroblasts

**Fig. 4:**
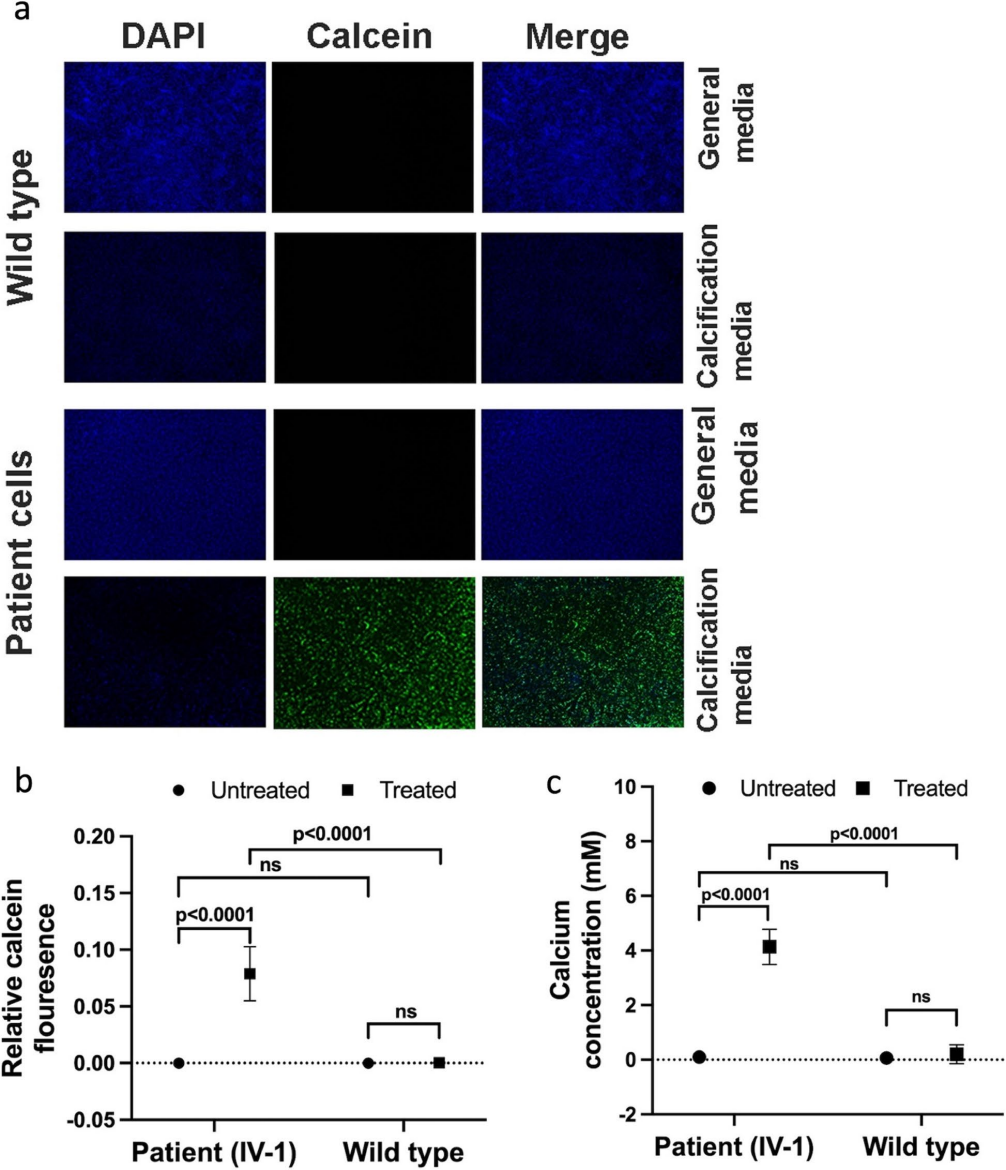
in vitro calcification assay in dermal fibroblast cells. **a** Confocal microscopy images show significant calcium phosphate deposits (Calcein fluorescence) in patient fibroblasts compared to wild type when supplied with pro-calcifying environment. **b** Calcein quantifications exhibit an increase calcium phosphate deposits in treated patient fibroblasts compared to treated wild type fibroblasts. **c** Graph shows a significant increase in calcium concentration in treated patient fibroblasts compared to treated wild type fibroblasts. Two-way ANOVA was utilized to assess the impact of standard and calcification medium on different genotypes i.e., patient and wild type

To assess the functional impact of this splice-site variant in *ENPP1*, the NPP (nucleotide pyrophosphatase/phosphodiesterase) enzymatic activity was measured in cell lysate from the patient and WT control. The patient fibroblasts exhibited an 87% reduction in ENPP1 enzymatic activity compared to the WT (*p* < 0.0001). The decrease in enzymatic activity was 7% more than the GACI control included in the experiment, which showed an 80% reduction compared to the WT control (Fig. 3c). The significant reduction in ENPP1 enzymatic activity suggests dysregulation of calcification as a likely cause of the cardiovascular phenotype. To test this, an in vitro calcification assay was performed to characterize mineral deposits by fluorescence microscopy and calcium quantification. Under the microscope, fibroblasts from both untreated patient and the healthy control sample showed negligible calcium phosphate deposits (Fig. 4a). When quantified by python script, both samples had a relative calcein fluorescence value of 0.0 (Fig. 4b), indicating that the samples were not calcified. However, in the treated fibroblasts, calcification in patient began as small and rounded precipitates, which gradually expanded to larger areas of mineralization. After 23 days of culture in calcification medium, the calcium phosphate deposits were spread throughout the cellular layer and the experiment was stopped. In contrast, the treated WT control sample showed negligible calcium phosphate deposits (Fig. 4a). Quantification showed a relative calcein fluorescence value of 0.08 for the patient fibroblasts, whereas the WT control had a relative fluorescence value of 0.0 (*p* < 0.0001) (Fig. 4b). Additionally, calcium levels were measured in the patient and WT control fibroblasts. In the untreated cells, there was no difference in calcium levels (both at 0.1 mM). However, in the treated samples, there was a significant difference in calcium levels (4.1 mM in patient vs. 0.2 mM in control, *p* < 0.0001) (Fig. 4c). (Text to be deleted: μM of substrates hydrolysis per μg of protein per hour: ENPP1.

## Discussion

ENPP1, which hydrolyzes ATP to generate AMP and inorganic pyrophosphate (PPi), is of interest due to its critical role in the regulation of soft tissue calcification and bone formation [7], and its wide spectrum of clinical presentations caused by their pathogenic variants [5]. Pathogenic variants underlying ENPP1 deficiency, particularly GACI, can occur throughout the ENPP1 protein. However, studies have shown that approximately 50% of disease-causing variants are located in the PC domain, while 27.1% are found in the NL domain [39]. The NL domain is strongly associated with the PC domain and plays a crucial role in the stability and enzymatic activity of the PC domain [40, 41]. Loss-of-function variants in either of these domains, therefore, are hypothesized to disrupt the enzymatic activity of ENPP1, resulting in low PPi levels and subsequent pathological calcification [22, 42].

The present study delves into the molecular mechanism of the novel homozygous splice site variant (c.2230 + 5G > A) in exon 21 of the *ENPP1*. This variant disrupts the intrinsic base sequence within the highly conserved sequence motif (AG/GUAAGU) at the 5′ splice sites (5′ss), potentially affecting base pairing with the core sequence of U1 small nuclear RNA (snRNA) within the spliceosome U1 snRNA, thus impacting the splicing process [43]. Sequence variations at intronic 5′ss positions can alter base-pairing strength, consequently affecting splicing outcomes such as exon skipping or cryptic splice site activation near the bona fide splice sites. Activation of a cryptic splice site usually results in the skipping of part of the adjacent exon or inclusion of part of the adjacent intron [44]. In silico analysis of the *ENPP1* variant c.2230 + 5G > A using the CRY*P-*SKIP algorithm predicts an 84% probability of exon 21 skipping and a 16% probability of activating a cryptic splice site. Experimental validation through PCR and Sanger sequencing of patients cDNA confirms exon 21 skipping. Notably, aberrantly spliced transcripts with cryptic splice site activation were not detected, suggesting that the variant primarily leads to exon skipping rather than cryptic splice site activation. It is likely that some transcripts underwent alternative splicing fate that were not detected on the electrophoresis [45]. As a consequence, the splice site variant leads to skipping of exon 21, resulting in premature termination of translation (p.Asp701Asnfs*2) and the deletion of about one fourth of the ENPP1 protein. The premature stop codon likely triggers the nonsense-mediated decay (NMD) pathway, causing degradation and preventing translation of the variant.

Further confirmation through western blot analysis demonstrates a loss of ENPP1 protein (Fig. 3b). The variant exhibits a damaging effect in functional studies, showing significantly reduced enzymatic activity of ENPP1 in patients fibroblasts leading to elevated calcification. This substantiates a plausible connection between the identified genetic variant and GACI, a condition arising from deficiencies in PPi, subsequently leading to abnormal buildup of calcium in the walls of the blood vessels, including those that carry blood from the heart to the rest of the body. The observed decline in ENPP1 activity in the proband closely aligns with that of another GACI patient utilized as a disease control during the experiment. Unfortunately, it was not possible to investigate the effect of the reduced ENPP1 enzyme activity on the PPi plasma level of the patient, as we did not have the opportunity to measure PPi due to logistical problems. Considering that ENPP1 deficiency is a well-established cause of GACI type 1 in infants, and arterial stenosis is one of the hallmarks of the disease, we attribute the patient’s cardiovascular phenotype to GACI. Around 72% of patients with GACI develop myointimal hyperplasia and/ or arterial stenosis, which might be independent of plasma PPi levels, but is dependent on AMP and further adenosine generation of ENPP1 [46]. The diagnosis of GACI could also provide an explanation for recurrent maternal abortions. A high frequency of spontaneous abortions, approximately 24% compared to the general population rate of 1–2%, has been observed in families with GACI [22, 45, 47]. This association underscores the importance of considering GACI in the context of recurrent pregnancy loss. The early onset of arterial or cardiovascular calcification and intimal proliferation has previously been linked to recurrent pregnancy loss, further emphasizing the relevance of this genetic variant in understanding the recurrent pregnancy loss phenomenon [17].

A total of 46 *ENPP1* variants have been reported, including nonsense, frameshift, missense, splicing variations, and large deletions [48]. GACI was first described in medical literature in 1899, and since then, over 250 cases with *ENPP1* variants have been reported [39, 49, 50]. The clinical presentation of GACI varies, with intra-familial and inter-individual phenotypic variability [22]. Our patient presents with the pulmonary stenosis and mild right ventricular hypertrophy, without a documented history of arterial calcification or heart failure. It is possible that the patient may develop the risk of hypophosphatemic rickets at a later stage, as many GACI patients with biallelic pathogenic variants in the *ENPP1* gene progress to develop ARHR2 [22, 51].

In summary, we described that how a novel splice site variant (c.2230 + 5G > A) in the C-terminal nuclease-like domain (NLD) of ENPP1 has a deleterious effect on the stability and function of the protein. This study emphasizes the crucial role of genetic analysis in accurate diagnosis, management, and counseling of affected individuals due to the disorder’s diverse and nonspecific symptoms. Furthermore, identifying this novel variant in the *ENPP1* gene expands our knowledge of the genetic landscape and mutation spectrum associated with GACI, offering insights into its pathogenesis and clinical management.

## Supplementary Information

Supplementary Material 1.

Supplementary Material 2.

## Data Availability

The variant discussed in this manuscript is deposited in the ClinVAR database with the accession number: VCV002584779.1. All other data generated or analysed during this study are included in this article (and its supplementary information files).

## Data availability

No datasets were generated or analysed during the current study.
