# Supplementary material for: Homozygous splice-site variant in ENPP1 underlies generalized arterial calcification of infancy
Source: BMC Pediatr. 2024 Nov 13;24:733. doi: 10.1186/s12887-024-05123-0 (PMC11558987; doi:10.1186/s12887-024-05123-0)
Supplement: Supplementary file 1 — Supplementary Material 1. [file 12887_2024_5123_MOESM1_ESM.docx]

**Supplementary Information:**

**Table S1. Primer sequences, related to Sanger validation, recovery of splicing defect and Real-time quantitative PCR**

| **Name** | **Forward sequence** | **Reverse sequence** |
| --- | --- | --- |
| *ENPP1*-DNA | TCAGGACAGTTTCTCTACGGA | ACATCAACTACCCTCCCAACA |
| *ENPP1*-cDNA | TCCCAGCACCAGTTTATGAGT | GTGTCTAGGTTTTCACAGTGCA |
| *ENPP1*-qPCR | TCTTCTTTCCCAGCACCAGT | CTGTCATTTCTGTCCACGGT |

**Table S2: List of homozygous variants within the ROH regions ≥5 Mb**

| **Chr:region (build GRCh38/hg38)** | **Gene** | **Mut cDNA**  **(Mut Prot)** | **MedPred** | **gnomAD**  **Frequency** | **dbSNP annotation** | **CADD**  **PHRED**  **V1.3** | **Poly-phene**  **2** | **SIFT** | **disease association**  **(OMIM number, mode of inheritance)** |
| --- | --- | --- | --- | --- | --- | --- | --- | --- | --- |
| 6:136359983 | *MAP7* | c.1807A>G (p.Lys603Glu) | 0.67 | 0.000008058 | rs766211439 | 25.2 | 0.81 | 0.01 | NA |
| **6: 131882479** | ***ENPP1*** | **c.2230+5G>A** | **0.91** | **-** | **-** | **14.46** | **0.01** | **0.05** | **(125853, AD; 601665, AD, AR;208000, AR; 615522, AD; 613312, AR)** |

**Supplementary table 2:** These variants are located within regions of homozygosity which are derived from exome sequence data. The CADD_phred, SIFT, PolyPhen, and MedPre scores show the *in-silico* pathogenicity prediction of the variant. Variant highlighted in bold represent the causative variant for the phenotype under investigation. Designations include NA (not applicable), AR (autosomal recessive), and AD (autosomal dominant). *ENPP1* resides in the the ROH region (GRCh38; chr6: 124,935,243- 143,850,264, size 18.9 Mb).


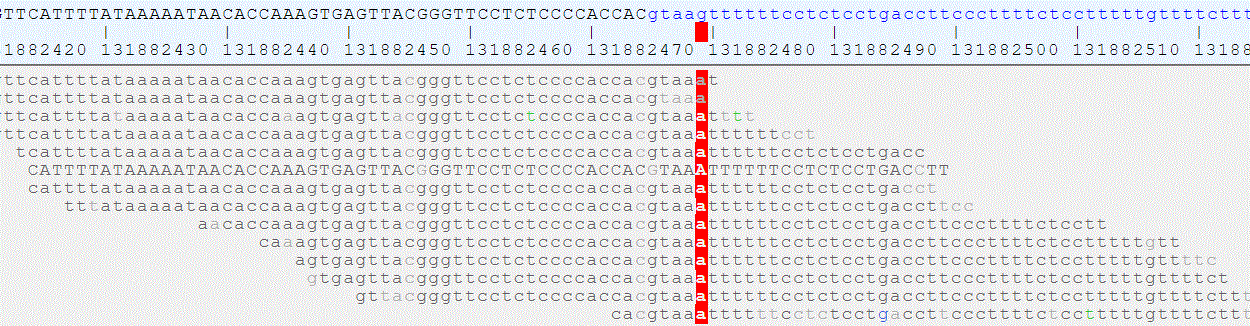


**Figure S1.** WES reads shows an *ENPP1* variant. Reads from the patient indicate a homozygous G-to-A transition at position +5 of intron 21 (https://varbank.ccg.uni‐koeln.de/varbank2/). Above, in black capital letters and small lowercase blue letters, are the genomic reference sequence and the hg38 coordinates. Below is the next-generation sequencing read alignment, where uppercase and lowercase letters correspond to exon and intronic regions, respectively. The genetic alteration is labeled in red.

**Online Web Resources**

gnomAD, ([https://gnomad.broadinstitute.org](https://gnomad.broadinstitute.org/))

1000 genome, ([http://browser.1000genomes.org](http://browser.1000genomes.org/))

dbSNP155, (<https://ftp.ncbi.nlm.nih.gov/snp/>)

CADD Phred score, (<http://cadd.gs.washington.edu/>)

VARBANK pipeline, (https://varbank.ccg.uni‐koeln.de/varbank2/)

Online Mendelian Inheritance in Man (OMIM), ([www.omim.org](http://www.omim.org))

Ensembl Variant Effect Predictor, (https://www.ensembl.org/Tools/VEP)

ClinVar, (<http://www.ncbi.nlm.nih.gov/clinvar>)

Human Gene Mutation Database, (<http://www.hgmd.org>)

dbNSFP, (http://database.liulab.science/dbNSFP#database)

MutationTaster, ([http://www.mutationtaster.org](http://www.mutationtaster.org/))

SIFT, ([https://sift.bii.a-star.edu.sg](https://sift.bii.a-star.edu.sg/))

PolyPhen‐2, (<http://genetics.bwh.harvard.edu/pph2>)
