# Supplementary figures and images for: Homozygous splice-site variant in ENPP1 underlies generalized arterial calcification of infancy
Source: BMC Pediatr. 2024 Nov 13;24:733. doi: 10.1186/s12887-024-05123-0 (PMC11558987; doi:10.1186/s12887-024-05123-0)

**Supplematary information:**

Full uncropped Gels and Blots images

**Figure 2b**

**
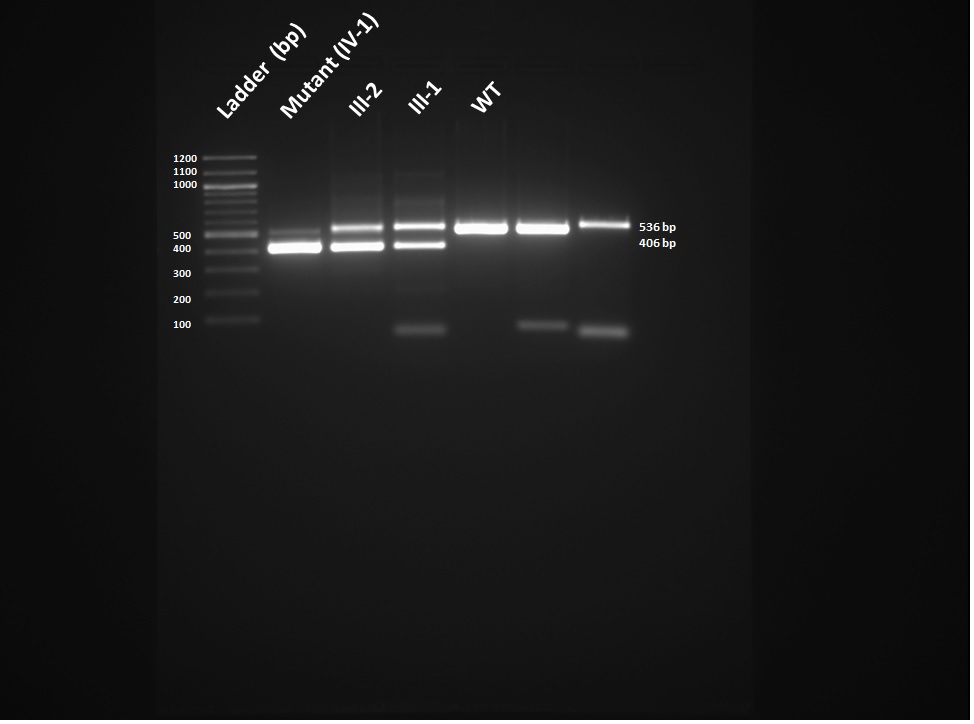
**

**Figure 3b**


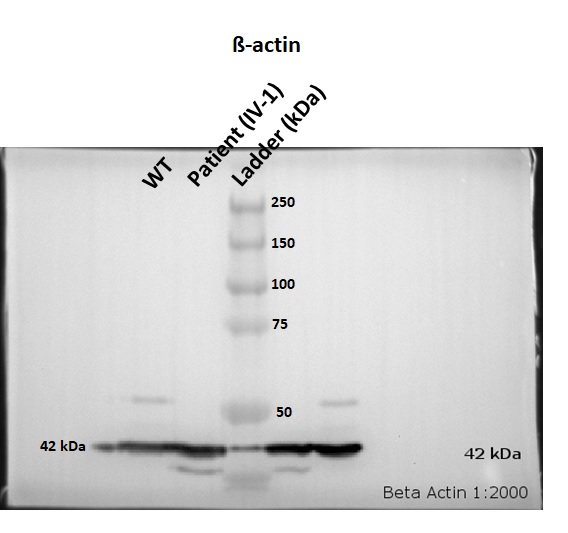


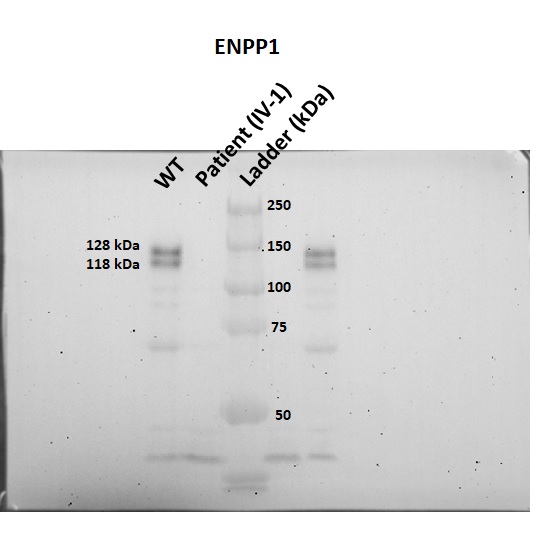

Supplement: Supplementary file 2 — Supplementary Material 2. [file 12887_2024_5123_MOESM2_ESM.docx]
